# Optical Imaging of Pancreatic Innervation

**DOI:** 10.3389/fendo.2021.663022

**Published:** 2021-04-27

**Authors:** Madina Makhmutova, Alejandro Caicedo

**Affiliations:** Division of Endocrinology, Diabetes and Metabolism, Department of Medicine, University of Miami Miller School of Medicine, Miami, FL, United States

**Keywords:** innervation, pancreatic ganglia, parasympathetic, sympathetic, vagus, nodose ganglia (ng), fluorescence imaging

## Abstract

At the time of Ivan Pavlov, pancreatic innervation was studied by looking at pancreas secretions in response to electrical stimulation of nerves. Nowadays we have ways to visualize neuronal activity in real time thanks to advances in fluorescent reporters and imaging techniques. We also have very precise optogenetic and pharmacogenetic approaches that allow neuronal manipulations in a very specific manner. These technological advances have been extensively employed for studying the central nervous system and are just beginning to be incorporated for studying visceral innervation. Pancreatic innervation is complex, and the role it plays in physiology and pathophysiology of the organ is still not fully understood. In this review we highlight anatomical aspects of pancreatic innervation, techniques for pancreatic neuronal labeling, and approaches for imaging pancreatic innervation *in vitro* and *in vivo*.

## The Status Quo of Pancreatic Innervation Is Still Based on Early Findings

First accounts of pancreatic innervation go back to the times of Paul Langerhans, who described pancreatic islets as richly innervated clusters of cells (1869), Santiago Ramon y Cajal, who delineated the innervation of the exocrine acinar tissue (1891), and Ivan Pavlov, who showed the role of innervation in pancreas secretion (1887) (1). An understanding of pancreatic innervation came quite far by the first half of the twentieth century. A paper published in 1945 already gave a detailed description of parasympathetic, sympathetic, and afferent innervation, as well as its potential targets and physiological effects in both endocrine and exocrine pancreas (2). Most of the postulates in that review came from studying the effects of neuronal stimulation on endocrine and exocrine secretion and anatomical studies of neuronal degeneration, which were confirmed later with more modern techniques, such as tracing, immunohistochemistry, and electrophysiology.

Although our understanding of innervation patterns, neuronal chemical phenotype, innervation targets, and interspecies differences has advanced over time, conceptually it does not go far beyond of what was known in 1945. The scientific and clinical community still perceives pancreatic innervation as a classical autonomic network of robust competing sympathetic-parasympathetic efferent inputs and a still mysterious afferent sensory branch. The intricate details of peripheral transduction mechanisms, as well as the big picture of the neural circuitry of the pancreas and its role in the physiology of endocrine and exocrine pancreas are still elusive. Modern techniques of optical imaging have answered many questions in the neuroscience field and brought our understanding of neural circuity and connectivity in the brain to a completely different level. This review discusses approaches and challenges of structural and functional optical imaging of pancreatic innervation.

## Structural and Functional Features of Pancreatic Innervation

**Figure 1:**
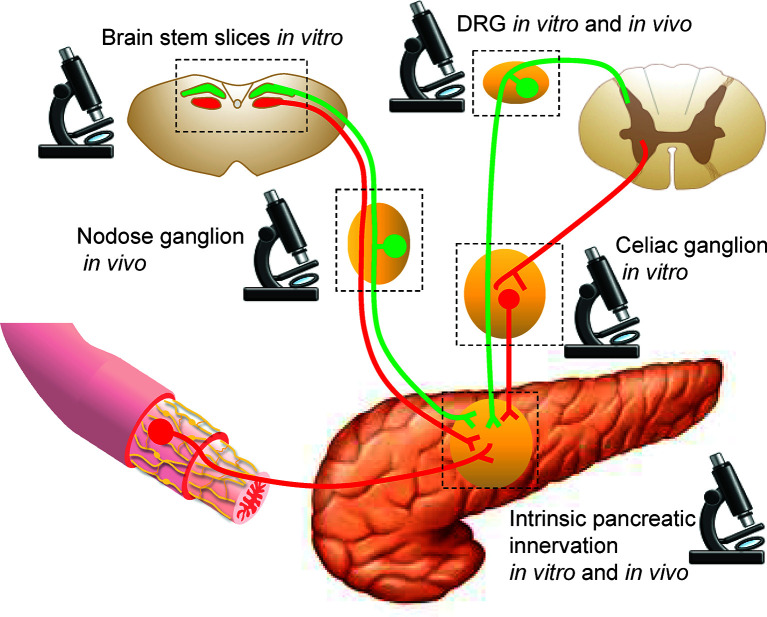
Sites for imaging pancreatic innervation. Schematic representation of extrinsic sources of pancreatic innervation that converge on intrinsic pancreatic ganglia. Microscope icons indicate imaging sites.

As the details of pancreatic innervation have been reviewed elsewhere (3–7), here we briefly go over some structural and functional features of innervation relevant for imaging, with an emphasis on neuroanatomical circuitry and neuronal phenotypes. The most intuitive way to segregate pancreatic innervation for practical imaging purposes is by the location of the neuronal soma: inside (intrinsic) or outside (extrinsic) of the pancreas (**Figure 1**).

### Intrinsic Pancreatic Innervation

**Figure 2:**
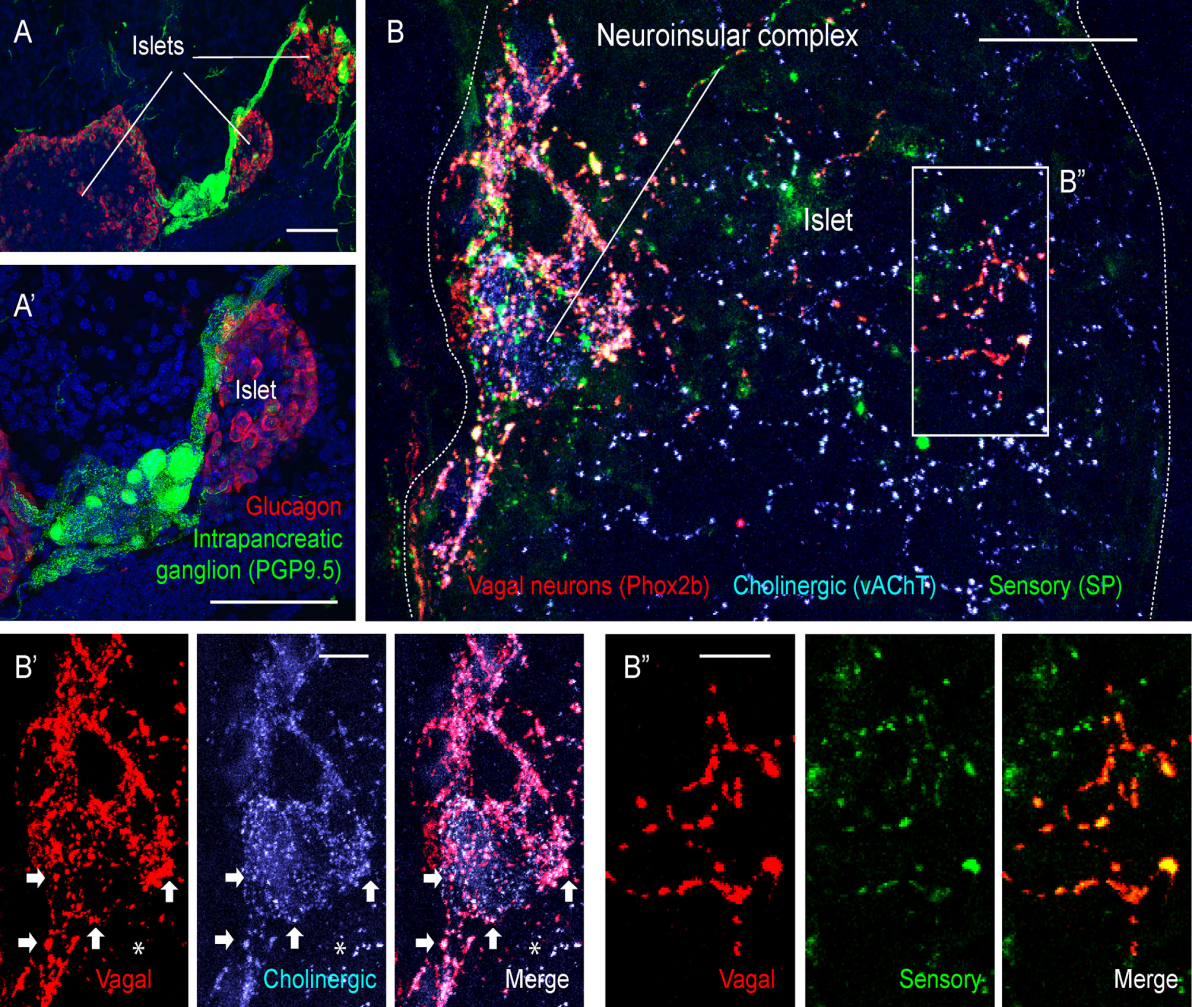
Extrinsic and intrinsic cholinergic innervation of the endocrine pancreas (figure adapted and modified from Supplementary Figures 4, 5 in Makhmutova et al, 2020). **(A, A’)** Mouse pancreatic section immunostained for the pan-neuronal marker PGP9.5 (green) and glucagon (red); scale bar 50 µm. **(B, B’’)** Pancreatic section of a Phox2b-Cre-tdTomato^floxed^ transgenic mouse, immunostained for red fluorescent protein (red), the sensory neuronal marker substance P (SP, green), and the cholinergic neuronal marker vesicular acetylcholine transporter (vAChT, blue). Inside the neuroinsular ganglion, Phox2b-positive varicosities colocalize with vAChT (**B’**, *arrows*), indicating terminals of vagal preganglionic neurons; scale bar 20 µm. Outside of the neuroinsular ganglion (inside the islet), Phox2b-positive fibers colocalize with SP **(B”)** but not with vAChT (**B’**, *asterisks*), indicating vagal origin of sensory fibers and non-vagal origin of intrinsic cholinergic fibers; scale bar 5 µm.

The intrinsic pancreatic innervation is a network of pancreatic neurons that cluster to form pancreatic ganglia and send unmyelinated projections to their targets in the endocrine and exocrine pancreas (8). Ganglia are dispersed throughout the pancreatic parenchyma and interlobular spaces, but are also present around some pancreatic islets, where they form neuroinsular complexes (9, 10). Anatomical heterogeneity and interspecies differences have been reported for pancreatic ganglia. The physiological significance of these differences, however, remains elusive (9, 11). Although the majority of pancreatic neurons is immunoreactive for cholinergic markers (ACh, VAChT, ChAT) and are thought to be postganglionic parasympathetic neurons (5, 12), only a fraction of them actually receives primary parasympathetic input (12, 13). In addition to cholinergic parasympathetic input, pancreatic ganglia also receive input from noradrenergic sympathetic neurons, substance P (SP)- and calcitonin gene-related peptide (CGRP)- positive sensory neurons, and serotonergic and cholinergic enteric neurons (12, 14–18). Thus, intrinsic neurons in pancreatic ganglia represent neuronal hubs that integrate multimodal extrinsic neuronal input and send an integrated message throughout the pancreas primarily *via* unmyelinated cholinergic projections (**Figure 2**). The histological and electrophysiological properties of pancreatic ganglia and their responsiveness to neurotransmitters, endocrine, and paracrine substances have been studied thoroughly [for a review, see (3, 5, 6)].

Most cholinergic terminals observed throughout the pancreas are thought to be projections of intrinsic pancreatic neurons (**Figure 2**). These fibers travel in the perivascular space and branch out to innervate the vasculature, endocrine cells of pancreatic islets, exocrine acini, and the ductal system. Innervation of endocrine islets varies between species. In mouse islets, cholinergic fibers target α and β-cells, while in human islets endocrine cells are rarely contacted directly (9, 19). Interspecies differences raise many questions about postsynaptic targets, molecular mechanisms, and the physiological role of this innervation branch in the human endocrine pancreas.

Overall, stimulation of the intrinsic cholinergic system *via* multimodal extrinsic input is thought to activate the digestive state of the endocrine and exocrine pancreas by promoting vasodilation and increased secretion of insulin and exocrine enzymes. In parallel to its “rest and digest effects”, cholinergic innervation is also an important player in mediating inflammatory responses in exocrine and endocrine pancreas (20, 21).

### Extrinsic Pancreatic Innervation

Extrinsic neural input into the pancreas is composed of five autonomic branches: two efferent pathways – parasympathetic and sympathetic; two afferent pathways - vagal and spinal; and the entero-pancreatic axis. While primary parasympathetic neurons project exclusively to intrinsic ganglia, the other extrinsic branches also target pancreatic structures directly (12).

#### Parasympathetic Innervation

Primary parasympathetic neurons with cell bodies in the dorsal motor nucleus of vagus (DMV) (12) travel in the vagus nerve and synapse exclusively in the pancreatic ganglia without targeting acinar or endocrine structures directly (12). Both primary parasympathetic and intrinsic pancreatic neurons are cholinergic. These different neural populations have distinct embryological origins and can be distinguished by embryonic markers. Vagal neurons (both afferent and efferent) are derived from the neurogenic placodes and can be visualized and accessed using the placodal-specific Phox2b-Cre mouse line (**Figure 2**). The neural crest derived neurons (intrinsic pancreatic, splanchnic, and enteric) can be visualized and accessed using the neural crest-specific Wnt1-Cre line (22, 23). It is generally assumed that parasympathetic innervation brings strong excitatory input to the intrinsic pancreatic neurons, presumably *via* nicotinic cholinergic receptors (13, 16, 24–30). This input is intensely modulated by abundant extrinsic pathways at the level of the ganglia (31–35). Activation of the parasympathetic system facilitates the secretion of digestive enzymes and glucose-lowering insulin and is thought to be responsible for preparing the organ for an increased digestive demand during the cephalic phase (24, 28, 36–42).

#### Sympathetic Innervation

**Figure 3:**
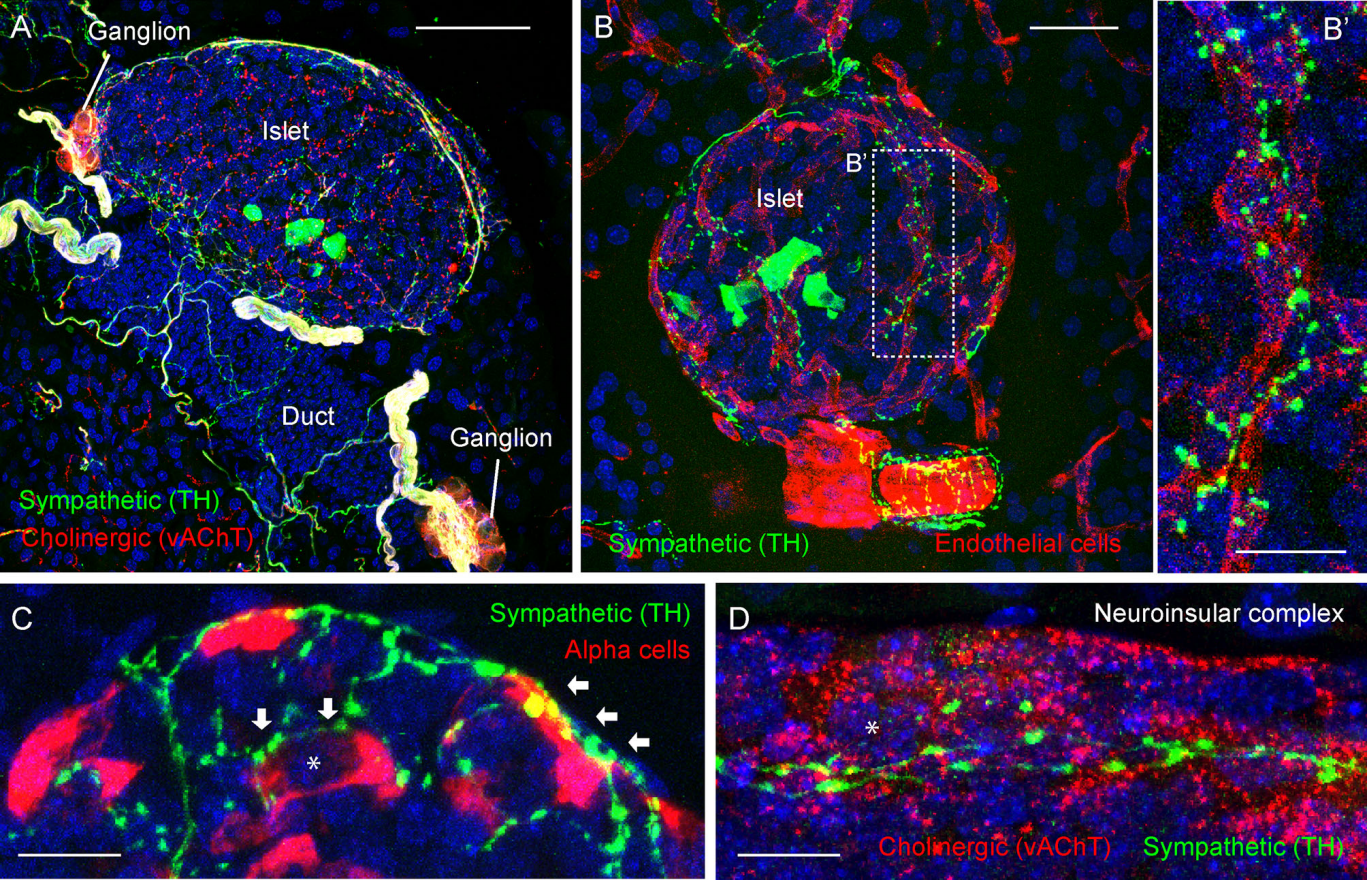
Adrenergic innervation of the endocrine pancreas (photomicrographs are from experiments conducted by Dr. Joana Almaca, unpublished data). **(A)** Mouse pancreatic section immunostained for the sympathetic marker tyrosine hydroxylase (TH, green), the cholinergic marker vesicular acetylcholine transporter (vAChT, red), and DAPI (blue); scale bar 50 µm. **(B-B’)** Mouse pancreatic section immunostained for TH (green), endothelial marker CD31 (red), and DAPI (blue); scale bars 20 µm **(B)** and 10 µm **(B’)**. **(C)** Mouse pancreatic section immunostained for TH (green), glucagon (red), and DAPI (blue); scale bar 10 µm. **(D)** Mouse pancreatic section immunostained for TH (green), vAChT (red), and DAPI (blue); scale bar 10 µm. TH-positive fibers can be seen along the perivascular space of a large blood vessel **(B)** and intra-islet capillaries **(B’)**. TH-positive varicosities contact endothelial vascular cells **(B’)**, endocrine α-cells (*arrows* in **C**), and neurons within a neuroinsular complex **(D)**. Asterisks denote an alpha cell (in **C**) and a neuron (in **D**).

Primary sympathetic neurons reside in the intermediolateral column of the spinal cord and send projections to postsynaptic neurons of the perivertebral column and celiac ganglia *via* splanchnic nerves (36, 43). Noradrenergic projections of secondary sympathetic neurons enter the pancreas *via* mesenteric nerves and innervate pancreatic ganglia, vasculature, endocrine islets, ducts, and lymph nodes (**Figure 3**). Sympathetic activation leads to vasoconstriction, reduces exocrine secretion, and shifts endocrine secretion to the hyperglycemic state by lowering insulin and increasing glucagon levels (44, 45). In addition to this canonical sympathetic effect on pancreatic secretory function, several recent studies report an important interaction of sympathetic innervation with the pancreatic immune system (14, 46, 47), which could be involved in the development of autoimmune diabetes (48).

#### Sensory Innervation

The pancreas receives both vagal and spinal sensory innervation. Vagal sensory neurons have cell bodies in the nodose ganglion and their projections travel alongside parasympathetic efferent fibers in the vagus nerve. Spinal sensory neurons originating bilaterally in the T9-T13 dorsal root ganglia travel alongside sympathetic fibers in splanchnic and mesenteric nerves. Substance P- and CGRP-positive sensory fibers are found throughout exocrine tissue and in the majority of pancreatic islets. Vagal sensory fibers project preferentially to pancreatic islets, indicating that the exocrine sensory component is primarily of spinal origin (**Figure 2**), although a contribution of spinal afferents to islet innervation has also been reported (49–51). Pathologies of the exocrine tissue such as cancer and pancreatitis are usually perceived as very painful and associated with increases in sensory innervation (52, 53). Diabetes and insulinomas, by contrast, seem to develop without significant sensory awareness. Moreover, vagal afferents in the pancreas are chemosensors, while spinal afferents are preferentially mechanosensitive (14, 54). This supports the idea that vagal and spinal sensory pathways carry different sensory modalities: the vagal being more of homeostatic nature, while the spinal being more nociceptive. A small percentage of intrinsic pancreatic neurons in mice and human pancreas are substance P-positive, suggesting that the intrinsic pancreatic innervation might contain a sensory component. However, its sensory properties and physiological role remain elusive (11).

#### Enteric Innervation

The least studied and perhaps most intriguing neuronal input to the pancreas comes from the gut and is referred to as entero-pancreatic innervation. Tracing studies show that the neuronal plexus of the stomach antrum and proximal duodenum extends to the pancreas, sending cholinergic and serotonergic projections preferentially to intrinsic ganglia, and more sparsely to the acinar tissue, endocrine cells, vasculature, and ducts. While it is difficult to distinguish enteric cholinergic input from vagal parasympathetic and intrinsic cholinergic fibers, serotonergic terminals in the pancreas seem to originate exclusively from the enteric nervous system. The effect of serotonergic innervation on pancreatic neurons is not straightforward since the latter express both excitatory [5HT3R (55)], and inhibitory [5HT1 (55, 56)], receptors and intraluminal intestinal stimulation has been shown to elicit both excitatory (17, 57) and inhibitory (31) effects on pancreatic neurons and pancreas secretion. The enteric neuronal input to the endocrine pancreas has not been studied yet.

Intrinsic pancreatic neurons not only receive neuronal input from the gut (58), but also share anatomical similarities and embryonic origin with the enteric nervous system (8, 59, 60). Throughout her early work, Kirchgessner has been advocating for a continuity of the pancreatic network with the enteric nervous system (8, 59, 60). Moreover, vagotomy and sympathectomy have little effect on intrinsic pancreatic innervation (2), suggesting some degree of autonomy of the entero-pancreatic network from the central nervous system.

## Challenges for Studying Pancreatic Innervation

In their early work on pancreatic innervation, Rudolf Heidenhein, Claude Bernard, and Ivan Pavlov acknowledged that studying pancreatic physiology and especially its innervation is by far a more challenging task than studying the innervation of other secretory glands (1, 61). They described the pancreas to be highly sensitive to surgical manipulations and experimental results to be extremely variable. Pavlov explained some of the experimental variability as a result of complex antagonistic interactions of innervation circuits and neuronal sensitivity to experimental conditions. Indeed, intrinsic pancreatic innervation receives multimodal input from all possible extrinsic innervation sources. Thus, output from intrinsic cholinergic neurons is a cumulative response to all extrinsic inputs, not a classical parasympathetic pathway. This is further complicated by the effects of extrinsic innervation on cardiovascular, exocrine, and endocrine systems, by the central nervous system feedback triggered by afferent innervation, and by local neuronal reflexes of the entero-pancreatic innervation.

The pancreas performs two very distinct yet essential functions: production of digestive enzymes by the exocrine compartment and secretion of the glucoregulatory endocrine hormones by its endocrine compartment. The pancreatic innervation is thus designed to control both exocrine and endocrine functions and target very distinct cell types. This heterogeneity, however, is very difficult to disentangle and make sense of.

The majority of axonal terminals inside the pancreas are non-myelinated axons that are difficult to visualize. For instance, in their work on sympathetic innervation of the pancreas, Alm et al. noticed that sympathetic terminals around small capillaries were very thin (under 0.2 um) and contained unusually low levels of catecholamines (at least 100 fold lower of what has been observed in other tissues), making visualization of such fibers only possible after catecholamine-increasing treatment with L-Dopa or dopamine (62). To improve visualization of axonal terminals, a wide range of transgenic reporter mice is now available along with advanced viral neuronal tracing techniques.

Neuronal fibers in the pancreas do not form classical synapses with well-defined pre- and post-synaptic structures. Instead, they contain neurotransmitter-rich varicosities along their path and release their contents into the surrounding space affecting nearby target cells with appropriate receptors. This makes innervation effective in its ability to affect multiple targets simultaneously and introduces another level of physiological complexity but hinders the identification of postsynaptic targets and physiological effects.

Despite a wide body of literature on pancreatic innervation, many physiological and even anatomical questions remain unanswered and need to be addressed. Modern neuronal labeling and imaging techniques introduce means for overcoming many technical challenges and tackling those questions (**Table 1**).

**Table 1:** Open questions in the field of pancreatic innervation.

Innervation Branch	Open questions in the field
Intrinsic pancreatic innervation	Differences in postganglionic cholinergic innervation of rodent and human endocrine pancreas raise many questions about postsynaptic targets, molecular mechanisms, and the physiological role of this innervation branch in different species. The role of neuroinsular complexes and how they differ from other intrinsic ganglia that are not directly associated with endocrine islets remains unknown. The anti-inflammatory effect of cholinergic innervation on resident macrophages has been proposed, but not confirmed.
Parasympathetic	Although the vago-vagal reflex has been proposed to be involved in the regulation of glucose metabolism, the parts of the circuit have not been linked together. It has not been determined if the parasympathetic efferent output depends on vagal sensory input from the pancreas. Hypothalamic and higher brain centers control of parasympathetic innervation in glucose metabolism is not fully understood. The anti-inflammatory properties of parasympathetic innervation have been described systemically as well as in many visceral systems, but has been barely studied in the pancreas.
Sympathetic	Adrenergic neurons innervate pancreatic islets and draining lymph nodes and are suspected to be important players in the pathogenesis of autoimmune diabetes. The anatomical, physiological, and molecular properties of the interactions between sympathetic neurons and the local components of the immune system requires further research.
Enteric	Extensive tracing studies suggest that the enteric nervous system extends projections into the pancreas. The physiological significance of the entero-pancreatic network, however, is poorly understood.
Sensory	Sensory transduction mechanisms and sensory modalities transmitted via spinal and vagal afferents are not well understood. Although systemic manipulation of sensory innervation affects glucose metabolism, it is still not clear if and how pancreas-specific sensory pathways affect glucose metabolism and exocrine secretion, and whether the vagal sensory component is involved in the mediation of the vago-vagal reflex. The etiology and pathogenesis of chronic pain in pancreatitis and pancreatic cancer is widely studied, but still not fully understood. Sensory innervation is proposed to be an important mediator in pathogenesis of pancreatic cancers.

## The Arsenal of Fluorescent Tools to Study Pancreatic Innervation

A significant research effort focuses on developing non-invasive imaging approaches with the use of magnetic waves, ultrasound, and radiation. These techniques, unfortunately, are not sensitive enough to visualize peripheral innervation. Fluorescence microscopy is one of the most informative experimental approaches in neuroscience today as it has capacity not only for structural visualization but also for functional imaging and optical manipulation of delicate neuronal structures. Limited light penetration, however, requires tissue isolation or quite invasive *in vivo* preparations that restrict this technique to the experimental niche. Here we will discuss fluorescent neuronal labeling and existing approaches of optically accessing intrinsic and extrinsic pancreatic innervation.

The neuroscience field offers a wide variety of tools for neuronal labeling. These include antibody-dependent techniques, genetically encoded reporters, and neuronal dyes for structural and functional imaging (**Tables 2**–**4**). While most neuronal markers are shared between different neuronal types, several reporters allow to distinctively label major peripheral branches of innervation: cholinergic (vAChT, ChAT), adrenergic (TH), and sensory (SP, CGRP, TRPV1) fibers (**Table 2**). Some neurons can further be distinguished by placodal or neural crest embryonic origin markers (**Table 2**). Sequencing studies of peripheral neuronal ganglia identify unique markers for peripheral innervation providing deeper molecular and genetic characterization for various neuronal types (63, 64). These markers are then used as guides for developing neuronal antibodies and generating transgenic reporters of selective neural pathways.

**Table 2:** Neuronal markers, antibodies, mouse transgenic lines, and promoters for neuronal labeling.

Innervation type	Marker	Antibodies validated in the pancreas	Promoters/Transgenic lines
Cholinergic (primary parasympathetic, primary sympathetic, intrinsic pancreatic)	ACh		N/A
	vAChT	139103, Synaptic Systems	SLC18A3-cre (PMID: 14502577)
	ChAT		ChAT-eGFP (Jax 007902) ChAT-Cre (Jax 006410)
Adrenergic (secondary sympathetic)	TH	AB152, Millipore	TH-Cre (Jax 008601)
Peptidergic-sensory (vagal afferents, spinal afferents, intrinsic sensory)	SP	MAB356, Millipore	Tac1-Cre (Jax 021877)
	CGRP	BML-CA1134, Enzo	CALCA-Cre/eGFP (Jax 033168)
	TRPV1	Alomone labs ACC-030	TRPV1-Cre (Jax 017769)
	5HT3R		5HT3R-GFP (PMID 19095802) 5HT3R-Flpo (Jax 030755)
	Pirt		Pirt-GCaMP3 (JHU C13628) Pirt-Cre (JHU C13783)
Serotonergic (enteric)	5HT		N/A
Placodal-derived (vagal afferent and efferent)	Phox2b		Phox2b-Cre (Jax 016223)
Neural Crest-derived	Wnt1		Wnt1-Cre (Jax 022137)
Pan-neuronal markers	b3Tub	MRB-435P, Covance	TUBB3-eGFP (MGI:4847518)
	Pgp9.5	#233003, Synaptic Systems	UCHL1-eGFP (Jax 022476)
	Thy1		Thy1-Cre (Jax 006143)
	Snap25		Snap25-GCaMP6 (Jax 025111)
	Synaptophysin (Syp)		Syp promoter RC::FPSit (Jax 030206) Ai34D (Jax 012670)
	Synapsin (Syn)	Cell Signaling Technology 5297 #106011C5, Synaptic Systems	Syn1-Cre (Jax 003966) hSyn - most common AAV promoter on AddGene
	Acetylated tubulin	T6793, Sigma	TUBA1A promoter
	NeuN		RBFOX3 promoter
	S100	ab52642, Abcam	S100b-Cre (Jax 014160)
	NF200	Sigma-Aldrich N4142	
	cFos	#226003, Synaptic Systems	TRAP2 (Jax 030323)

**Table 3:** Examples of Cre-dependent reporter mouse lines for neuronal labeling.

	Reporter line	Stock number	Use
Structural reporters	ROSA26-eGFP	Jax 004077	eGFP reporter
	Ai6	Jax 007906	Ubiquotous expression of ZsGreen1 reporter throughout dendrites and long axons
	Ai14	Jax 007914	Ubiquotous expression of tdTomato reporter throughout dendrites and long axons
Functional reporters	Ai95	Jax 028865	Ca^2+^ indicator GCaMP6f (fast response kintetics)
	Ai96	Jax 028866	Ca^2+^ indicator GCaMP6s (slow response kinetics)
	TRAP2	Jax 030323	Cre-dependent reporter expression in active neurons expressing immediate early gene cFos
Neuro-modulators	Ai32	Jax 012569	Channelrhodosin-2/eYFP fusion protein for optogenetic stimulation
	RC::FPDi	Jax 029040	Inhibitory DREADD-mCherry fusion protein for pharmacogenetic inhibition
	RC::L-hM3Dq	Jax 026943	Excitatory DREADD-mCherry fusion protein for pharmacogenetic stimulation

**Table 4:** Conventional dyes for neuronal labeling.

	Neuronal dye	Labeling properties	Cell loading
Cytoplasmic	Hydrazides and Biocytins	Intracellular bidirectional tracer Assesment of gap junctions	enter cells by microinjection and iontophoresis, may cross gap junctions
	Dextran conjugates	Intracellular bidirectional tracer	enter cells by microinjection and through resected neuronal terminals
Membrane-bound	Choleratoxin subunit B (CTB)	membrane-bound bidirectional tracer	Binds to plasma membrane glycosphingolipids
	Lectins (WGA, Phaseolus Vulgaris, DBA, GS)	membrane-bound bidirectional tracer have limited trans-synaptic labeling capacity (WGA)	Bind various plasma membrane carbohydrates
	DiI	Lateral diffusion in plasma membrane Bidirectional tracer Can be used in live or fixed tissue	Lipohilic dye, incorporates into lipid bilayer
Functional	AM and FM dyes	Non-fluorescent in aqueous solution, become fluorescent upon internalization	Enter cells by endocytosis or through non-selective ion channels Internalization can be activity-dependent
	Calcium indicator dyes (Fluo-4, Fura-2)	Become fluorescent upon Ca^2+^ binding, Activity-dependent	Esterified dyes are membrane permeable, trapped inside cells after cytoplasmic de-esterification
	Voltage-sensetive dye (FluoVolt, ANEP)	Become fluorescent upon changes in electrical potential	Bind plasma membranes (FluoVolt), membrane-permeable (ANEP)

Antibody dependent neuronal labeling is a classical immunohistochemical approach for structural delineation of neuronal pathways. It is, however, limited by antibody specificity, tissue penetration, and epitope availability. While few neuronal markers are abundantly expressed (Pgp9.5, b-Tubulin) throughout a neuron, localization of most neuronal epitopes can vary along the neural process (i.e. axon). Thus, antibodies do not label neuronal shafts uniformly and do not always allow delineating fine neuronal projections and terminals. Although labeling of living non-permeabilized neurons with antibodies against neuronal surface markers has been reported, it is very uncommon and only suitable to homogeneous neuronal cultures (65). Neuronal antibodies are preferentially suited for histology in fixed tissue.

Neurons can be targeted specifically by driving expression of fluorescent reporter proteins or transcriptional drivers under a neuron-specific promoter. This provides flexible access to neuronal populations for structural and functional imaging and for neuronal manipulations. Transgenic animal models that drive expression of either Cre-recombinase, fluorescent reporters, or Ca^2+^ indicators are available for the most common neuronal promoters (**Tables 2**, **3**). Work with transgenic animals, however, requires validation of gene expression specificity since transgene expression does not always reflect endogenous promoter activity. Wonderful reviews summarize currently existing transgenic tools for targeting central and peripheral innervation (66, 67). Transgenic labeling is widely used for structural imaging because it allows efficient labeling of neuronal projections that greatly complements antibody-dependent neuronal labeling. To allow physiological recording of neuronal activity *in vivo* and in living pancreatic slices, neuronal promoters and Cre-drivers can be coupled to expression of the genetically encoded calcium indicator GCaMP. Analogously, transgenic expression of optogenetic (channelrhodopsin), chemogenetic (DREADD), and magnetogenetic (TRPV1-ferritin) receptors provides access to neuronal manipulation.

Viral delivery of transgenes allows targeting neurons specifically with improved spatial and temporal resolution (68). Pancreatic neurons can be transfected by injecting the virus into the pancreatic parenchyma (69), infusing the pancreas with the virus through the common bile duct (70), or by less specific injections into extrinsic neuronal ganglia. Viruses are excellent for long range neuronal tracing and neuronal manipulation *in vivo* and can potentially be used with pancreatic slices *in vitro.* Because pancreatic tissues are very heterogeneous, neuronal specificity of viral transfection requires the use of neuron-specific or Cre-dependent viral vectors and viral particles with increased neuronal tropism.

Being replaced by transgenic animal models and viral tracing approaches, conventional neuronal dyes are unjustly placed on the sideline of neuroscience research today. Compared to transgenic reporters, neuronal dyes are relatively inexpensive and allow acute neuronal labeling across different species, which can be especially advantageous for assessing innervation in human tissues (e.g. in living pancreas slices from human pancreatic donors). Cytoplasmic and membrane dyes are useful tools for structural assessment of innervation and can be used for long-range neuronal tracing *in vivo*, local neuronal labeling in living pancreatic tissue slices, and even in fixed tissues (in the case of DiI, **Table 4**). Functional neuronal dyes allow to monitor neuronal activity either by binding intracellular signaling molecules (Ca^2+^ and cAMP indicators), responding to changes in electrical potential (membrane voltage dyes), or by activity-dependent internalization of the dye (AM and FM dyes, **Table 4**). The majority of conventional dyes, however, have limited neuronal specificity and thus must be applied locally.

## Structural Imaging of Innervation in Fixed Pancreas Tissues

In the field of islet biology, the most common histological approach for studying endocrine cells involves widefield imaging of thin tissue sections (<10 μm). This, however, is not suitable for studying neuronal projections that with elaborate three-dimensional terminal trees. Thicker pancreatic cryo-sections (40-60 μm) allow sufficient antibody penetration, excellent access to confocal imaging, and easy histological processing on the slide without harsh clearing protocols (**Figures 2**, **3**). Confocal imaging of thick pancreatic cryo-sections allowed delineating parasympathetic and sympathetic innervation, reconstructing axonal terminals in 3D in human and mouse pancreatic tissues with excellent spatial resolution, and identifying their potential postsynaptic targets (19, 22).

The now popular technique of organ clearing allowed whole pancreas imaging and 3D reconstruction of entire pancreatic innervation. This technique is especially informative in understanding innervation continuity and heterogeneity throughout the whole pancreas and gives a better perspective on connectivity between endocrine and exocrine compartments (9, 11, 71–74). While advantageous for studying neuronal connectivity and histology of large neuronal structures such as neuronal ganglia and large neurites, this approach is not always adequate for analyzing fine neuronal terminals and their postsynaptic targets due to limited subcellular resolution. Moreover, tissue clearing involves use of harsh organic compounds which may interfere with epitope preservation, and usually requires lengthy incubations (days to weeks).

## Live Imaging of Pancreatic Innervation

Live imaging of innervation requires making target tissues visually accessible without significantly interfering with tissue physiology. Intrinsic pancreatic innervation and extrinsic neuronal terminals can be imaged at the level of the pancreas either in the intact pancreas or in living pancreatic slices. Extrinsic pancreatic innervation can also be imaged outside of the pancreas in corresponding extrinsic ganglia. Innervation of pancreatic islets can be studied longitudinally after transplantation into the anterior chamber of the eye. To avoid all the hurdles of exposing innervation in rodent models, pancreatic innervation can be studied in transparent animal models such as zebra fish. And finally, islet innervation can be studied in artificial setting of co-culture of pancreatic islets and primary neurons.

### *In Vivo* Imaging of Intact Pancreas (Exteriorized Pancreas and Intravital Window)

The pancreas can be partially exteriorized and imaged acutely in anesthetized animals (46, 75–77). For gaining longitudinal optical access to the pancreas, several recent studies report the use of an intravital window installed into the abdominal wall. Unlike a very stable cranial window attached to the scull, the abdominal window is attached to the muscle, is more prone to movement and requires additional fixation of the pancreas to stabilize the field of view. Pancreas exteriorization and abdominal window provide access for imaging superficial structures at cellular resolution. Although innervation has never been imaged at the level of the intact pancreas *in vivo*, several studies report effective imaging of pancreatic endocrine cells, immune cells, and vasculature (46, 75–77), suggesting that this approach can potentially be used for imaging superficial large neuronal structures such as pancreatic ganglia and neuroinsular complexes. However, due to limitations of optical tissue penetration and residual tissue movement, this technique is not ideally suited for imaging fine neuronal projections and terminals.

### Living Pancreatic Slices

**Figure 4:**
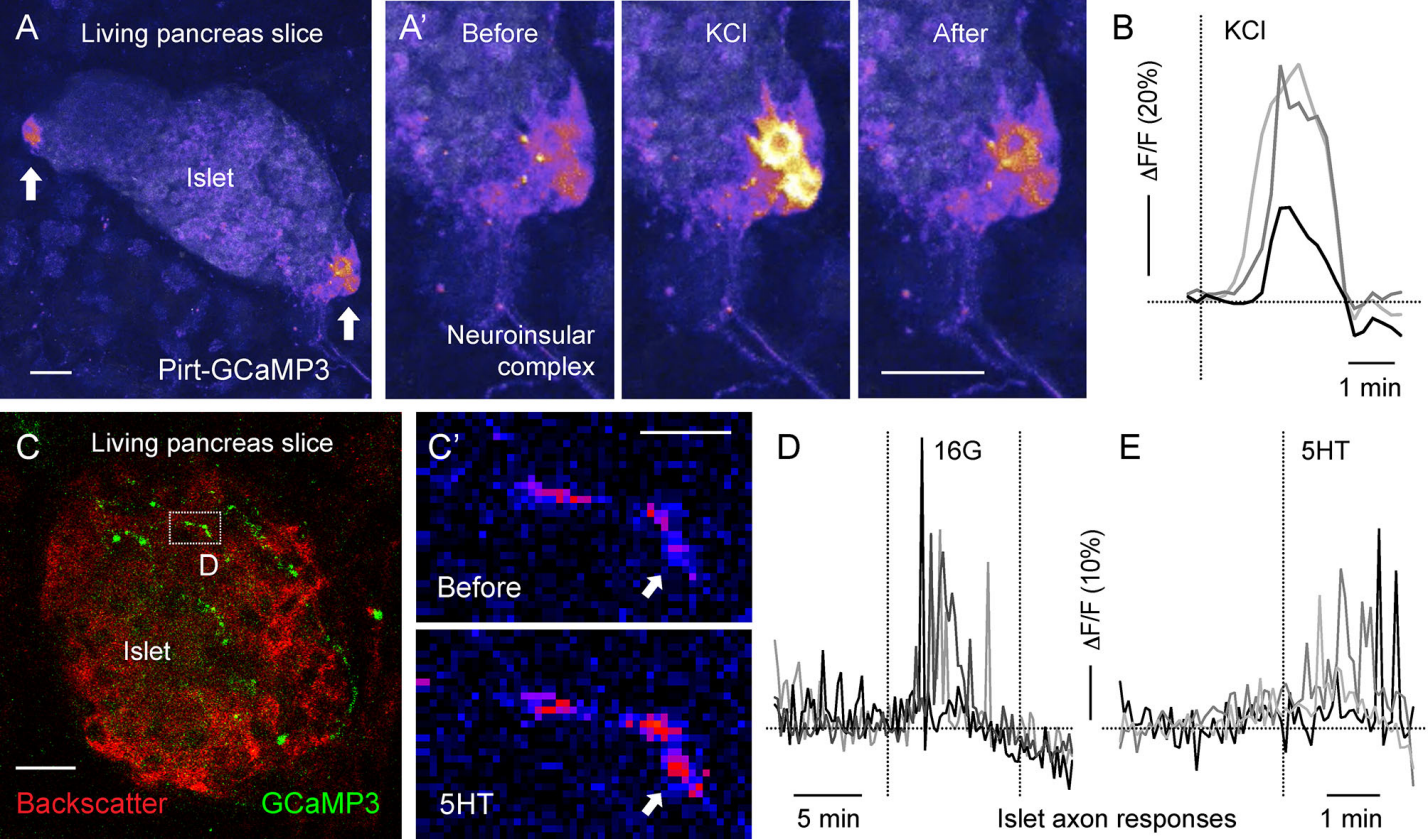
Living mouse pancreatic slices for imaging Ca^2+^ activity in neuroinsular ganglia and in sensory axonal terminals [photomicrographs are from experiments conducted by Dr. Jonathan Weitz, **(A, B)** unpublished data, **(C–F)** adapted and modified from Figure 6 in Makhmutova et al, 2020]. **(A, C)** Z-stacks of confocal images of living pancreatic slices from Pirt-GCaMP3 mice. **(A)** A representative islet containing neuroinsular ganglia (*arrows*), with GCaMP3 fluorescence and islet backscatter shown in pseudocolor scale; scale bar 10 µm. (A’) Sequential images of the neuroinsular ganglion shown in A, displaying a Ca^2+^ response to KCl (25 mM). **(B)** Representative traces of mean fluorescence intensity changes over baseline (δF/F) in the neuroinsular ganglion shown in **(A’)**, demonstrating neuronal responses to KCl stimulation. **(C)** Representative islet containing GCaMP3-expressing sensory axonal terminals (green). Islet backscatter is shown in red; scale bar 20 µm. **(C’)** Sequential images of the sensory fiber shown in C (box), displaying a Ca^2+^ response to 5-HT (50 µM). The *arrow* points to a region showing an increase in GCaMP3 fluorescence (pseudocolor scale); scale bar 5 µm. **(D, E)** Representative traces of mean fluorescence intensity changes over baseline (δF/F) in sensory fibers, demonstrating responses to an increase in glucose concentration from 3 mM to 16 mM (16G, **D**) and to 5-HT (50 µM) stimulation **(E)**.

Mouse and human pancreatic tissue slices are becoming a common tool for studying endocrine, exocrine, and vascular physiology (78–81). Living pancreatic slices provide optical access to deep pancreatic structures with a partially preserved native microenvironment. Slices with a thickness of 120-150 um can be almost entirely visualized with confocal microscopy, allowing high resolution imaging of fine neuronal terminals [**Figure 4** (14)]. Many neuronal terminals in the slice, however, end up being cut from their neuronal soma and therefore might maintain their responsiveness for a short period of time or change their physiological properties. Moreover, labeling efficiency of fine neuronal projections in the pancreas remains problematic. In our experience, animals expressing a knock in GCaMP3 gene under the Pirt promoter have strongly labeled pancreatic terminals (**Figure 4**), while terminals could not be visualized in Pirt-Cre-GCaMP6 mice (14). Despite these challenges, this approach has proven itself effective for imaging various pancreatic cell types, including neuronal terminals, and holds a lot of potential for functional imaging of pancreatic innervation.

Functional imaging of living tissue slices from human donors is an invaluable resource for studying physiology of human pancreas. Although functional studies of human pancreatic innervation have not been reported yet, we assume it is possible but challenging due to difficulties in neuronal labeling and the high neuronal vulnerability to ischemia with its associated anoxia.

### Eye Model for Studying Islet Transplants

**Figure 5:**
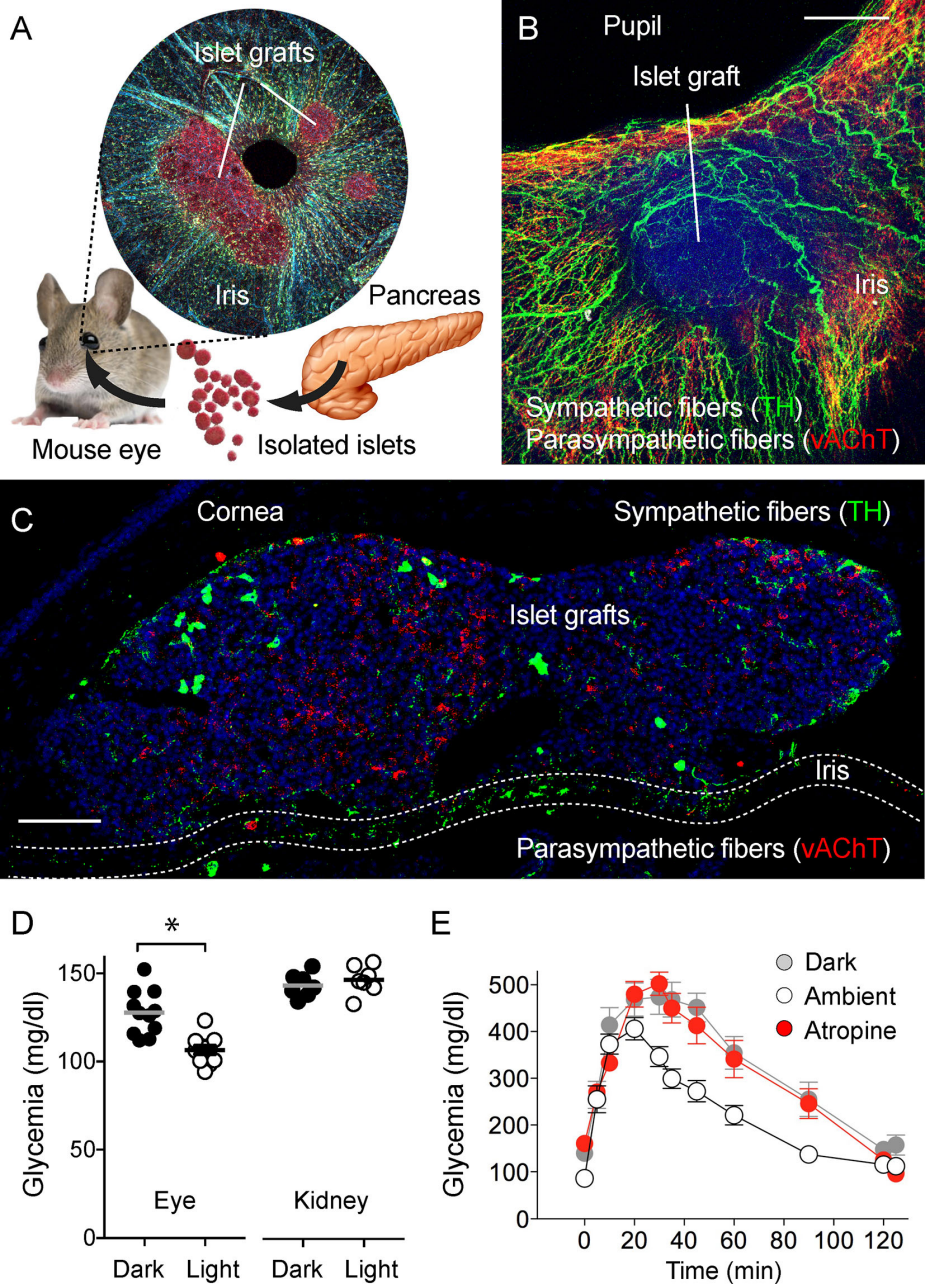
Eye model for imaging innervation of pancreatic islet grafts [photomicrographs are from experiments conducted by Dr. Rayner Rodriguez-Diaz (**A–C**, unpublished data], data in **(D)** and **(E)** adapted and modified from Figure 4 in Rodriguez-Diaz et al., 2012). **(A)** Schematic representation of islet transplantation into the anterior chamber of the eye. Islets isolated from the pancreas are transplanted into the mouse eye. Microphotograph of an iris wholemount with islet grafts. **(B)** Z-stack of confocal images of a wholemount of the mouse iris with an islet graft immunostained for TH (green) and vAChT (red); scale bar 50 µm. **(C)** Confocal image of a cross-section of the mouse eye showing two islet grafts, stained for TH (green), vAChT (red), and DAPI (blue); scale bar 50 µm. **(D)** Plasma glucose levels in mice transplanted with islets in the anterior chamber of the eye or under the kidney capsule, in ambient light or in the dark (*p < 0.05, paired Student’s t-test). **(E)** Glucose excursion during an IPGTT of transplanted mice performed in the dark (gray symbols), ambient light (open symbols), or in ambient light after topical application of the muscarinic receptor antagonist atropine (red symbols).

A creative attempt that is complementary to imaging the intact pancreas and pancreatic tissue slices is the “eye model” of noninvasive longitudinal imaging of pancreatic islets (82, 83). In this approach, islets isolated from the donor (mouse or human) are transplanted into the anterior chamber of the eye of a recipient mouse. Islets engraft on the iris of the eye, vascularize and establish innervation (**Figure 5**). The eye, in this case, plays a role as a natural imaging chamber and allows noninvasive longitudinal imaging with minimal movement artifacts in head-fixed anesthetized animals. Interestingly, once engrafted, the iris’ autonomic nerves innervate the islet. The newly developed islet innervation thus becomes dependent on the pupillary light reflex. When the light is on, parasympathetic stimulation triggers pupillary constriction and induces insulin secretion in engrafted islets. In dark ambient conditions, sympathetic activation triggers the pupil to dilate, and insulin secretion returns back to baseline levels (84). While the peripheral innervation of the islet graft and responses of the graft to neuronal stimulation are physiologically accurate, the neuronal pathways are not linked to the autonomic centers innervating the pancreas.

### Imaging Extrinsic Nuclei and Ganglia That Send Neuronal Projections to the Pancreas

Axonal terminals of extrinsic innervation can be studied at the level of the pancreas using the approaches listed above. Extrinsic innervation can also be examined at the level of the neuronal cell bodies that reside in brain nuclei or peripheral ganglia outside of the pancreas.

The dorsal vagal complex of the brainstem includes dorsal motor nucleus of vagus (DMV), composed of primary parasympathetic neurons, and the nucleus of the solitary tract (NTS), the region that receives sensory input from vagal sensory neurons. This site is believed to be an integral part of the vago-vagal reflex. Although the existence of this reflex has been confirmed for the exocrine pancreas (85), and has been proposed for the endocrine pancreas, a pancreas-specific pathway in the dorsal vagal complex remains to be described. Neurons of the dorsal vagal complex can be labeled by tracing from the pancreas (14, 69, 86) or by activity-dependent genetic labeling (87) induced by pancreas-specific stimulation. Once labeled, these neurons can be studied using imaging approaches *in vivo* or in brainstem slices (88, 89).

Celiac ganglia are the largest ganglia in the peripheral nervous system and are the major site of postganglionic sympathetic neurons innervating the pancreas (43, 90). Although sympathetic neurons have been extensively studied morphologically and electrophysiologically (91), a characterization of sympathetic subpopulations based on innervation targets is still lacking. Perhaps investigators were not motivated to do so because of the common notion that sympathetic stimulation induces a uniform inhibitory effect on digestion. Recent studies, however, are revealing an important interaction of sympathetic nerves with the immune system, particularly with immune cells of lymph nodes associated with the pancreas (14, 46, 47). A characterization of postganglionic sympathetic neurons therefore becomes physiologically and even clinically relevant. Although *in vivo* imaging approaches of the celiac ganglia have not been developed yet due to its inaccessible anatomical localization, explants of the ganglia can be studied morphologically and physiologically using immunohistochemical and calcium imaging approaches in combination with retrograde tracing from the pancreas.

**Figure 6:**
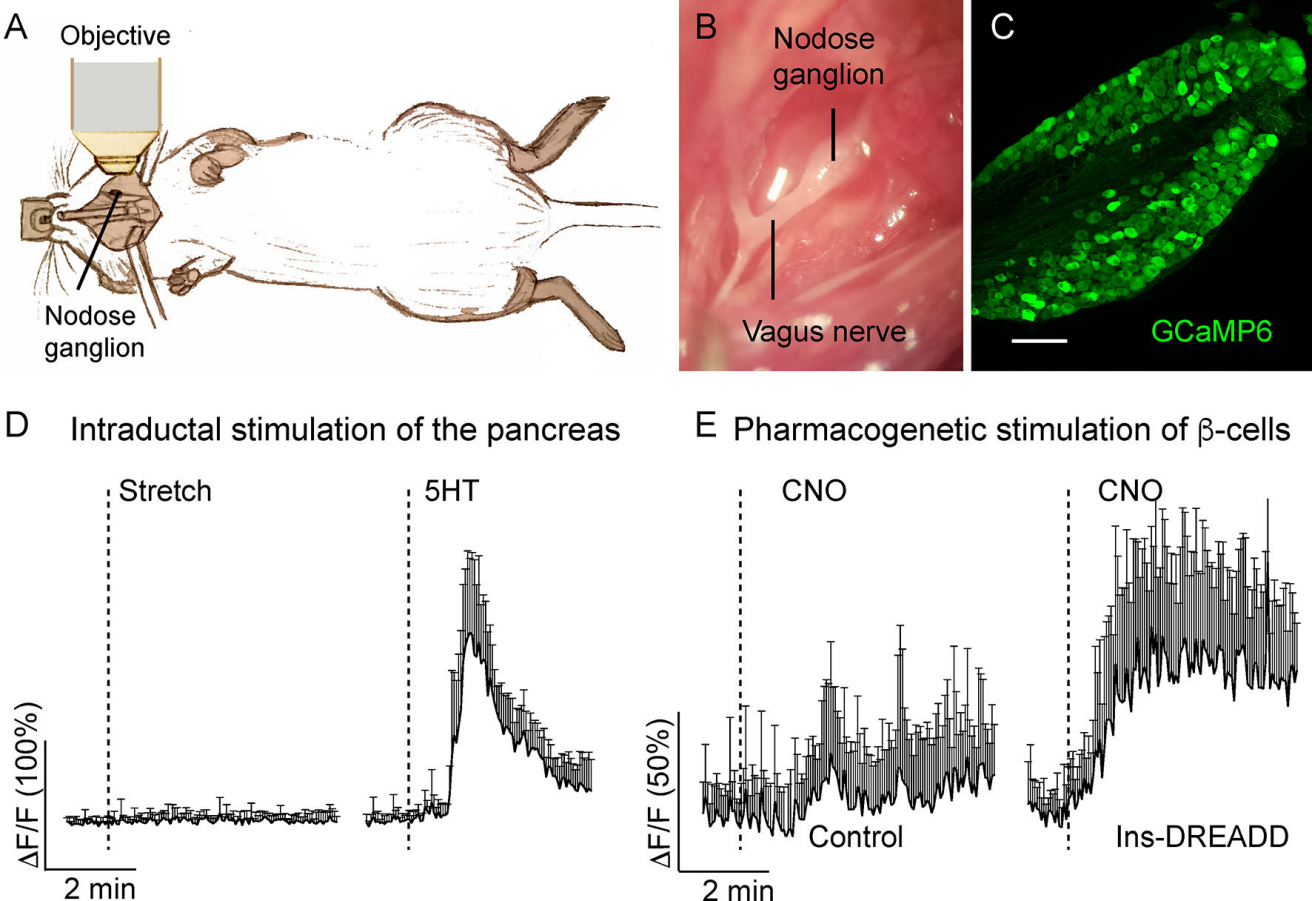
*In vivo* imaging of the mouse nodose ganglion (figure adapted and modified from Figures 3, 4 in Makhmutova et al, 2020). **(A)** Drawing illustrates the experimental set up, where the intact nodose ganglion is exposed for Ca^2+^ imaging under a confocal microscope. **(B)** Photomicrograph of the exposed nodose ganglion. **(C)** Section of the nodose ganglion from a Pirt-GCaMP6 mouse. **(D)** Average traces of fluorescence intensity changes over baseline (δF/F) in nodose ganglion neurons, demonstrating neuronal responses to stretch (intraductal pancreatic infusion with saline, 300 µl/min) or intraductal application of 5-HT (1 mM, 150 µl/min). **(E)** Average traces of fluorescence intensity changes over baseline (δF/F) in nodose ganglion neurons, demonstrating neuronal responses to pharmacogenetic stimulation of β-cells by i.p. injection of clozapine nitric oxide (CNO, 5 mg/kg) in control mice or in mice expressing designer receptor activated by designer drug (DREADD) exclusively in β-cells. Shown is the mean trace of 10 neurons (+/- SEM).

Nodose and dorsal root ganglia (DRGs) are respectively the sensory ganglia of the vagal and spinal sensory pathways that receive input from the pancreas (50). Because sensory innervation has been attributed a role in the maintenance of glucose homeostasis (49, 92, 93), decoding the signals at the level of the primary sensory neuron becomes an essential step in understanding the type of information the pancreas is transmitting to the brain *via* autonomic nerves. By recording activity of nodose ganglion neurons *in vivo*, we showed that these neurons are pancreatic chemosensors, responsive to selective stimulation of β-cells, presumably *via* serotonin-dependent mechanism, and are not sensitive to mechanical stimulation of the pancreas (**Figure 6**) (14) The sensory modality transmitted through the DRG remains to be discovered, but *in vivo* imaging of the DRG is technically challenging (94).

### *In Vivo* Imaging of Pancreatic Innervation in Zebra Fish (PMID: 29916364)

The zebra fish is an experimental model that has been used for studying multiple aspects of pancreas biology including pancreatic development, endocrine cell regeneration, vascularization and innervation (95–97). Its transparency during larval stages makes the zebra fish a great model for functional live imaging. Easy access for genetic manipulations allows efficient multi transgenic labeling of neurons and other pancreatic cell types simultaneously. Also, a short lifespan facilitates studies on development. Time-lapse *in vivo* imaging of zebra fish allowed delineating the sequence of events that leads to parasympathetic innervation of the pancreatic islet and measuring in real time changes in the endocrine cell mass in response to targeted neuronal ablation (97). Although advantageous for imaging pancreatic innervation and having multiple physiological and morphological similarities to the mammalian systems, processes learned in the zebra fish model need to be analyzed through the prism of interspecies differences.

### Co-Culture of Neurons and Pancreatic Cells

**Figure 7:**
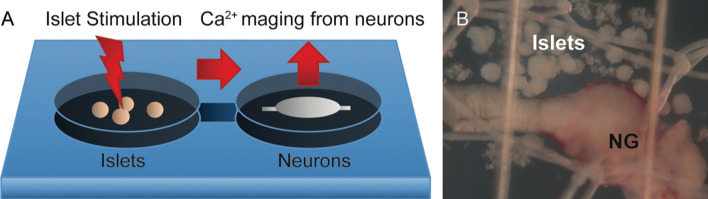
Potential model for recording Ca^2+^ neuronal activity in response to islet stimulation *in vitro*. **(A)** Schematic representation of the proposed experimental setup, where islets are placed upstream of neurons in a perfusion chamber or a microfluidics device. Pharmacological or optogenetic stimulation of islets will induce release of substances that have a potential to act on downstream neurons. **(B)** Representative photomicrograph of islets placed in close proximity to the nodose ganglion (NG) in an imaging chamber.

Studying synaptic transmission between a neurite and its postsynaptic target is a challenging task in the *in vivo* experimental setting. It is very difficult to access and manipulate the selected individual synaptic interaction. Furthermore, the local physiological mechanism cannot be uncoupled from the confounding systemic effects innervation has. The interactions between nerves and its targets can be better addressed *in vitro* (**Figure 7**). A beautiful example of synapse formation *in vitro* has been shown by culturing trigeminal sensory neurons with intestinal enteroendocrine cells (98). An example closer to islet biologist are studies showing that sympathetic neurons cultured with pancreatic islets triggered changes in islet cytoarchitecture inducing β-cell migration (99). Co-culture experiments might be challenging as culturing conditions need to accommodate the needs of very distinct cell types, and primary neuronal cultures are famous for being difficult to maintain *in vitro*.

## Concluding Remarks

Pancreatic tissues and their innervation have been studied for decades using classical histology in combination with electrophysiology. Those laborious methods are being replaced by modern optical imaging techniques combined with precise genetic cellular labeling. While the pancreas biology field has implemented the approaches needed to access the pancreas optically (e.g. exteriorized pancreas, living pancreas slices), the neuroscience field offers a variety of ready to use tools for neuronal labeling and manipulation (e.g. transgenic mice, genetically encoded reporters, optogenetics). Although the pancreas remains a difficult organ to study, we now can deploy an arsenal of tools that will allow us tackling many longstanding questions in the field of pancreatic innervation.

## Author Contributions

MM wrote the original draft of the manuscript. AC reviewed the original draft, edited and approved final version of the manuscript, and designed the figures. All authors contributed to the article and approved the submitted version.

## Funding

This work was supported by the Diabetes Research Institute Foundation and National Institutes of Health grants R56DK084321 (AC), R01DK084321 (AC), R01DK111538 (AC), R01DK113093 (AC), U01DK120456 (AC) R33ES025673 (AC) and R21ES025673 (AC), F31DK112596 (MM), the Leona M. and Harry B. Helmsley Charitable Trust grants G-2018PG-T1D034 and G-1912-03552.

## Conflict of Interest

The authors declare that the research was conducted in the absence of any commercial or financial relationships that could be construed as a potential conflict of interest.
